# Reversible Experiments: Putting Geological Disposal to the Test

**DOI:** 10.1007/s11948-015-9697-2

**Published:** 2015-09-12

**Authors:** Jan Peter Bergen

**Affiliations:** Section of Ethics/Philosophy of Technology, Faculty of Technology, Policy and Management, Delft University of Technology, P.O. Box 5015, 2600 GA Delft, The Netherlands

**Keywords:** Geological disposal, Reversibility, Retrievability, Social experiment, Lock-in

## Abstract

Conceiving of nuclear energy as a social experiment gives rise to the question of what to do when the experiment is no longer responsible or desirable. To be able to appropriately respond to such a situation, the nuclear energy technology in question should be *reversible*, i.e. it must be possible to stop its further development and implementation in society, and it must be possible to undo its undesirable consequences. This paper explores these two conditions by applying them to geological disposal of high-level radioactive waste (GD). Despite the fact that considerations of reversibility and retrievability have received increased attention in GD, the analysis in this paper concludes that GD cannot be considered reversible. Firstly, it would be difficult to stop its further development and implementation, since its historical development has led to a point where GD is significantly locked-in. Secondly, the strategy it employs for undoing undesirable consequences is less-than-ideal: it relies on containment of severely radiotoxic waste rather than attempting to eliminate this waste or its radioactivity. And while it may currently be technologically impossible to turn high-level waste into benign substances, GD’s containment strategy makes it difficult to eliminate this waste’s radioactivity when the possibility would arise. In all, GD should be critically reconsidered if the inclusion of reversibility considerations in radioactive waste management has indeed become as important as is sometimes claimed.

## Introduction

Ever since nuclear energy technologies were developed after the Second World War (WW2), we have been learning about the risks of nuclear energy production and how to deal with them.^1^ However, more than 50 years after nuclear power plants first supplied electricity to the grid,^2^ the Fukushima nuclear disaster made it excruciatingly clear that we are nowhere near done learning. Not only are there residual uncertainties about the risks of already widely deployed nuclear energy technologies, but new technologies are being developed (e.g., Generation IV reactors), while older ones have not seen widespread introduction even after decades of effort (e.g., geological disposal of radioactive waste).

However, how is this learning to be organized? The uncertainties and risks connected to nuclear power plant operation and radioactive waste management (RWM) have led van de Poel (2011) to propose that we should consider nuclear energy as a social experiment. This would mean that specific decisions on the acceptability of a technology, which now often occur before its actual introduction into society, would be replaced by an ongoing and conscious process of learning about its risks and benefits, as well as what is to be considered acceptable. So, understanding a nuclear energy technology as a social experiment would allow us to learn more about that technology’s risks and benefits as the experiment unfolds. Nonetheless, it also means that we might at one point learn what we, in a sense, would rather not, i.e., that continuing the experiment is no longer responsible or even that it is simply no longer desirable. What is an experimenter to do then? At the very least, she should be able to stop the experiment, and hazards should be contained as far as possible (van de Poel 2011).^3^ In earlier work (Bergen, in press) I contended that these two conditions, the ability to stop further development and implementation of a technology (the experiment) and undoing its undesirable consequences (e.g., hazards), are constitutive of technological *reversibility*. In other words, the technology experimented with should be reversible if the experimenter wants to be prepared for the experiment taking a turn for the worst.

This paper further explores what it means for a technology to be reversible by applying the abovementioned conditions for technological reversibility to a technology in which reversibility is already a salient consideration: the geological disposal of radioactive waste (GD). In doing so, the paper also provides an answer to whether or not GD can be considered reversible in the way required for responsible social experimentation.

## Reversibility as an Issue in Radioactive Waste Management

A quick exploration of publications by major nuclear organisations^4^ revealed five broad uses of the concepts of reversibility and irreversibility in the field of nuclear energy. The first three uses describe basic processes and consequences that are implicated in the production of nuclear energy:

- (Ir)reversible mechanical/chemical/thermodynamic processes during the production of nuclear energy or radioactive waste management (e.g., spent fuel reprocessing, drilling damage to repository host rock, and nuclear fission)
- A specific but important sub-category of the above: (ir)reversibility of flows and migrations, mostly of radioactive isotopes (e.g., in technical, environmental or geological systems). This aspect is often connected to standards for radioactive waste management facilities
- (Ir)reversibility of consequences, e.g.:
  - Mutations and cell damage in living tissue due to irradiation
  - Damage to the environment and its ecosystems

While these uses are useful for describing (ir)reversible aspects connected to nuclear energy, they are not actually oriented towards making a nuclear energy technology more reversible. However, the last two uses are oriented as such, since they provide specific design goals or strategies for reversible *radioactive waste management* (RWM) technology and its implementation:

- *Retrievability* of radioactive waste from a waste storage or geological disposal facility.
- *Reversibility* of (consequences of) decisions during the implementation process of a waste storage or geological disposal facility (e.g., Interagency Review Group on Nuclear Waste Management 1978; OECD Nuclear Energy Agency 2011; U.S. Department of Energy 1991)

Different RWM technologies differ in their plans for reversibility or retrievability of radioactive wastes, broadly determined by two factors. First, the type of radioactive waste is relevant. Generally, three categories of radioactive waste are distinguished based on their lifetime and radioactivity: low, intermediate, and high-level waste (IAEA 2009).^5^ High-level waste from nuclear energy production can be further divided based on the nuclear fuel cycle from which it results: it usually consists of either unprocessed spent nuclear fuel (SNF) or the still highly radioactive rest products of SNF reprocessing (HLW).^6^ The second relevant factor is the specific stage of RWM. For example, an interim storage facility has different ambitions for retrieving SNF or radioactive wastes than a final disposal site.^7^

For many low and intermediate-level waste, disposal and monitored storage (on- or near-surface) are considered realistic solutions until radioactive decay has rendered the wastes sufficiently unhazardous. Interim storage (on-surface, near surface or otherwise) for high-level waste^8^ is employed for (a) letting it decay and cool down to a point at which they become eligible for emplacement in a disposal facility, and/or (b) storing it until disposal facilities are available (Bonin 2010). In both cases, retrievability is an essential design feature. After such storage, however, a more permanent solution is generally deemed necessary for the further management of SNF and HLW, given the immense span of time that these materials remain potentially radiotoxic: geological disposal. A geological disposal facility, or repository, combines the protection offered by stable geological layers deep below the earth’s surface with multiple engineered barriers (e.g., overpack, clay, bentonite) around waste packages that contain either solid SNF or liquid HLW from reprocessing that has been stabilized in a confinement matrix (e.g., glass or concrete). All this is supposed to prevent radionuclides from reaching the human living environment until they have reached a safe level of decay (Bonin 2010). Given the time it takes for this level of decay to be reached, emplacement of SNF or HLW in a repository is, for all intents and purposes, meant to be indefinite.^9^ This solution supposedly allows the current generation to take responsibility for the radioactive wastes it produces, while not burdening future generations with it, nor counting on the longevity of institutions to maintain waste management practices for thousands of years.

Despite its *ultimate* goal of indefinite disposal of SNF and HLW for the reasons specified above, reversibility is increasingly recognized as a possibly important aspect of GD (e.g., Aparicio 2010; Weiss et al. 2013; OECD Nuclear Energy Agency 2011; Swedish National Council for Nuclear Waste 2010). Arguably, the most systematic proposal that describes how reversibility is supposed to feature in geological disposal has been put forward by the OECD Nuclear Energy Agency (NEA) as a result of their Reversibility & Retrievability project, in which it explored the role of reversibility considerations in GD. According to the NEA (2011):

- *Reversibility* “describes the ability *in principle* to change or reverse decisions taken during the progressive implementation of a disposal system […] The implementation of a reversible decision-making approach implies the willingness to question previous decisions in the light of new information, possibly leading to reversing or modifying them, and a decision-making culture that encourages such a questioning attitude” (p. 23; emphasis in the original).
- *Retrievability*, on the other hand, is “the ability *in principle* to recover waste or entire waste packages once they have been emplaced in a repository. Retrievability is the final element of a fully-applied reversibility strategy” (p. 24; emphasis in the original). Note that this does not mean that all high-level waste will also be practically accessible: past actions such as HLW vitrification might still exclude this possibility.

Both reversibility and retrievability apply here to the period before final closure of the repository, possibly up to 100 years after initial emplacement. Reversibility refers to a step-wise decision-making process, in which previous decisions can be undone. However, reversibility diminishes over time, as actions based on these decisions are partly cumulative and increase the costs and effort involved in undoing past decisions. Retrievability also gets more and more difficult as waste packages get sealed in place and the repository gets backfilled over time (OECD Nuclear Energy Agency 2011). Thus, final closure of the repository also means the end to a realistic possibility of reversibility and retrievability. Indeed, reversibility and retrievability are not considered to be “design goals” for GD. Rather, they are seen by the NEA as “attributes of the decision-making and design processes that can facilitate the journey towards the final destination of safe, socially accepted geological disposal” (OECD Nuclear Energy Agency 2012 p. 22). In other words, they are only instrumental in achieving the ultimate (design) goal of GD that has been set forward since its origins in the 1950s (e.g., National Research Council 1957)^10^: *passive safety*, or safety without human intervention. Still, a number of reasons are put forward as justifying the importance of reversibility and retrievability for GD:

- Reversibility would allow future generations to use the emplaced materials as a resource, especially since SNF contains plutonium and uranium which might have value as a future source of energy.
- Further technical advances might make it possible to render radioactive wastes (more) harmless.
- If a repository performs worse than expected, remedial action would be facilitated by reversibility provisions.
- Finally, reversibility can help foster public acceptance of waste disposal facilities, or help adapt waste management if public or policy attitudes change over time (OECD Nuclear Energy Agency 2011).

However, as Barthe (2010) points out: the goal of final disposal of wastes a century after initial emplacement as well as the regressive nature of reversibility and retrievability seem contradictory to these reasons for adopting reversibility in the first place. First of all, it will probably take a significant amount of time to develop technology for using a repository’s contents as resources or for making high-level waste less harmful. If this is the case, why would one want to have reversibility and retrievability diminish and possibly disappear before such technology can be developed and implemented on a sufficient scale? Secondly, repository performance becomes significantly more difficult to assure with increasing extrapolation into the long-term future. As such, reversibility and retrievability as a response to worse-than-expected repository performance has a higher change of becoming useful as time goes by. These considerations cast doubt on the extent to which GD could live up to the NEA’s own reasons for reversibility given above. On top of all this, it is clear that the choice of technology is a foregone conclusion in the NEA’s framework. It is concerned with how to implement a specific technology: GD. Yet, the recognition that changing public and/or policy attitudes towards RWM should be able to influence RWM strategies, as is shown in the fourth reason for reversibility, is of importance here. What if, for whatever reason, GD does not turn out to be the apt solution the technical community takes it to be (OECD Nuclear Energy Agency 1995),^11^ and/or democratic considerations would point us towards other technologies? Should our past decision for GD not also be reversible?

If GD reversibility provisions were, analytically speaking, fully in line with the reasons given for these provisions, these discrepancies should not exist. And yet, these discrepancies are here and warrant our attention. In this paper I would like to propose an outlook on technological reversibility that could (a) provide some insights in how technologies like GD become irreversible, (b) could help explain why the discrepancies above exist as they do, and (c) provide input concerning the way technologies like GD could be made more reversible. In so doing, I explore whether GD can actually be considered a *reversible technology*. If it can, then the above criticisms might be moot. However, it might also turn out that despite the efforts visible in the NEA’s Reversibility & Retrievability project, GD cannot be considered properly reversible. If so, we might need to reconsider either GD as the dominant high-level waste management technology or whether, why and to what extent we want reversibility in the first place.

To answer the question whether GD can be considered properly reversible, it is necessary to have an idea of what constitutes a reversible RWM technology. Elsewhere, I have argued that for a nuclear energy technology to be considered reversible, two conditions need to be both met:

- The ability to stop the further development and deployment of said technology in a society
- The ability to undo the undesirable outcomes of the development and deployment of the technology when so desired (Bergen, in press).

While arguably adequate as abstract descriptions of what constitutes ‘ideal’ reversibility, these conditions are not yet sufficiently operationalized to be useful in considering practical cases such as the one presented here. For one, their form does not yet invite either questioning or qualified answers. Secondly, they are not yet case-specific. As such, I would like to rephrase the conditions as two GD-specific questions that, if both answered affirmatively, would show that GD is reversible. These questions are:

1. *Would an authorized body be reasonably able to switch from GD to an alternative solution if problems with GD were to arise?* If not, the first of the conditions for reversible technology would not be met, and GD cannot be considered fully reversible.^12^
2. *Does GD exhaust the possibilities of undoing the consequences connected to high*-*level waste, and the hazards that could come about due to the use of GD for managing this waste?* Again, a negative answer to this question would disqualify GD as a reversible technology.

In what follows, I deal with these two questions in turn. In “On the Ability to Stop Further Development and Deployment of Geological Disposal”, the first question is examined by taking a closer look at the historical development of GD through the lens of path dependence and lock-in. I answer the second question in “On Geological Disposal’s Capacity for Undoing Consequences”, where I propose that the ability to undo undesirable consequences of GD is connected to the choice between different design strategies, and that GD’s chosen strategy is less-than-ideal.

## On the Ability to Stop Further Development and Deployment of Geological Disposal

In this section, the following question is considered: would an authorized body be reasonably able to switch from GD to an alternative solution if problems with GD were to arise? To answer this question, it is important to understand why switching to an alternative could become difficult or impossible in the first place. According to the theory of path dependence and lock-in, such difficulties can arise as a result of a historical process of technological development that leads to a situation in which switching to another solution becomes increasingly difficult: the technology becomes locked-in. As such, investigating the development of GD through the lens of path dependence and lock-in could help answer the question at hand. Before discussing GD’s historical development and whether it is locked-in or not, the theory behind path dependence and lock-in is briefly introduced below.

### Path Dependence and Lock-in

We call the development and implementation process of a specific technology path dependent if that process is determined by its own history (David 2007). That is, due to its specific characteristics, such a process can become inflexible in terms of the practical possibility of changing their course due to them being unable to “shake free from their histories” (David 2001 p. 19). Such path dependent processes, contain two main elements (Arthur 1989):

- A *contingent starting period*. This period is contingent in the sense that it does not originate in a smooth and predictable historical sequence of events but rather that a new element (e.g., the introduction of a new technology) sets history off on a novel path.
- A period exhibiting *‘increasing returns’*. Arthur identified four major types of increasing returns: scale economies, learning effects, adaptive expectations and network economies (Arthur 1994). While increasing returns can be conceived of quite narrowly as increasing efficiency, David (2007) considers it more appropriate to conceive of them as “self-reinforcing, positive feedback mechanisms governing decisions such as the choice among alternative production techniques, or consumer goods, or geographical locations for production activities” (David 2007). This self-reinforcement consists of both “positive and negative mechanisms that decrease the likelihood that alternative paths will be selected” (Vergne and Durand 2011). Positive mechanisms directly support the path (e.g. economies of scale or learning effects), while negative mechanisms operate by rendering alternative paths less interesting. As such, these mechanisms sustain the path that was contingently selected.

In some cases, this self-reinforcement can be so efficacious that it leads to an irreversible outcome, i.e., lock-in (Mahoney 2007; Vergne and Durand 2011). While initially options are open and multiple outcomes are possible, path dependence and self-reinforcement lead to (more and more) irreversibility that, without exogenous shock, could be incredibly persistent. If so, the potential for endogenous change becomes rather low (Mahoney 2007).

According to David (2007), one fundamental aspect of these self-reinforcing dynamics or increasing returns is the presence of micro-level irreversibilities, which occur when “a finite and possibly substantial cost must be incurred to undo the effects of the resource allocation decision in question” (David 2007 p. 101). So, these micro-irreversibilities make agents favour certain options for action, while disfavouring others due to the relative opportunities and costs involved in pursuing them. This effect is further strengthened if these micro-irreversibilities are interdependent, since it becomes less favourable to undo specific micro-irreversibilities if this requires undoing others as well. As such, they are constitutive of the self-reinforcement of dominant structures by guiding agents’ behaviour towards adherence to the most dominant (technological) solution, eventually strengthening its lock-in and increasing its irreversibility. Two notes about these micro-irreversibilities are in order. First, different types of lock-in seem to correspond to different sorts of micro-irreversibilities driving path-dependent processes. Indeed, different types of drivers of a technology’s lock-in can be found in the literature. For example, there is political (e.g., Walker 1999, 2000), institutional (e.g., Foxon and Pearson 2008; Walker 1999, 2000), economical (e.g. Arthur 1989; Liebowitz and Margolis 1995), and infrastructural (e.g., Frantzeskaki and Loorbach 2010; Scrase and Smith 2009) lock-in of a specific technology or technological project or system. While these have a different emphasis on what is most determinative of the lock-in in question, they all refer to sets of *symbolic*, *institutional* and/or *material* micro-irreversibilities that underlie the reinforcing dynamics. For high-level waste management, such micro-irreversibilities cover a spectrum of elements, from stabilization and packaging of HLW, test sites for GD and nuclear reactors producing SNF (material) to preferred methods of risk evaluation, nuclear regulations and policy prescriptions and practices, and institutional commitment (institutional), as well as underlying narratives, themes and values (symbolic). Secondly, it is interesting that non-material micro-irreversibilities could drive path-dependent processes. As such, even if a process of technology development results mainly in institutional or symbolic elements, a technology could (in theory at least) become locked-in without significant deployment of said technology in the real world as long as increasing returns are sufficient to keep actors committed to that technology. Finally, micro-irreversibilities lie at the basis of the positive and negative mechanisms that can lead to lock-in, i.e., make a technology practically irreversible. As such, this framework seems to combine both (ir)reversibilities *within* GD as micro-irreversibilities (which include the matters the NEA’s concepts of retrievability and reversibility is meant to address), as well as the (ir)reversibility of GD *itself* as a technology for radioactive waste management.

In what follows I will use the history of civil nuclear energy and high-level waste management in the USA between 1944 and 1987 as an example to show how GD’s development can exhibit the characteristics of a path-dependent process. While certainly not an exhaustive example [GD is held to be the appropriate solution in most nuclear energy-producing countries (U.K. Nuclear Decommissioning Authority 2008, 2013)], I hope it is sufficiently powerful for showcasing the type of historical process that lies at the basis of GD’s dominance. First, I present a sketch of GD’s contingent genesis in the years after WW2, after which I elaborate on its history from the late 1950s onwards.

### Geological Disposal’s Contingent Starting Period: Nuclear Development Between 1944 and 1957

The contingent starting period that set the stage for our current situation in which GD is the dominant solution for civil high-level waste management in the USA can be situated in the period between 1944 and 1957.

During WW2, nuclear development was dominated by military applications, both in weapons technology (developing the atomic bomb in the Manhattan project) as well as reactor design (producing plutonium for the weapons program). This dominance continued in the years after the war, one result of which was the development of the pressurized water reactor [PWR] for use in submarines^13^ (Cowan 1990). Given the circumstances of WW2, this initial focus on developing nuclear applications was rather straight-forward. For all that, these developments were prioritized over the careful and necessary management of the wastes they produced. While in 1944, the first HLW facility was constructed at the Hanford site in the State of Washington to store liquid HLW from the military nuclear program,^14^ many low- and intermediate level wastes were dealt with through ‘dilute and disperse’ strategies (Mckinley, Alexander and Blaser 2007; Miller, Fahnoe and Peterson 1954). This early focus on applications rather than proper waste management was further exacerbated by how the Atomic Energy Commission (AEC) was set up in 1946, being responsible for both the promotion as well as regulation of nuclear development. Its focus was much more on promoting the development of nuclear applications than on strict regulation, and secrecy ensured its control over nuclear matters (Clarfield and Wiecek 1984). In combination with relatively small waste volumes and the isolated location of the facilities at which this waste was produced, this led to RWM being basic (if not haphazard) until the early 1950s. In at least one sense, this was surprising: WW2 had graphically shown both the potency as well as the destructive capabilities of splitting the atom. In 1953, however, nuclear safety found strong political expression in Eisenhower’s ‘Atoms for Peace’ speech,^15^ which was to set the stage for the development of a peaceful civil nuclear energy program, separate from the military one. As Jasanoff and Kim (2009) argue, the speech was aimed at symbolically containing the atom’s destructive potential so graphically illustrated in Japan only a few years before. It was also aimed at containing international fear of the USA as a nuclear superpower, and was to open the way towards the exploitation of the atom’s peaceful applications. With ‘Atoms for Peace’, then, a strong theme of *containment* of the dangers connected to the atom lay at the basis of the nuclear energy industry. The speech also called for a private nuclear energy industry.^16^ This meant limited government influence in the new industry, and making investment in it interesting for private investors. With the 1954 Atomic Energy Act, the patenting of nuclear energy technologies was opened up, and secrecy was partly lifted so that private parties could use previously confidential technical knowledge to develop nuclear energy applications.

After ‘Atoms for Peace’, the nuclear energy industry indeed started to develop. Since the nuclear energy program was pressured by Eisenhower’s intentions into a mode of urgency, a reactor type was chosen with which significant experience had already been accumulated in the military program: the PWR (Cowan 1990). However, a final solution for the disposal of HLW had not yet been settled upon. An important step towards that goal was taken when, at the request of the AEC, the Committee on Waste Disposal of the National Research Council produced a report (National Research Council 1957) that would prove to be foundational for the development of GD and the values or aspirations it embodies. It argued that (after additional research), deep geological disposal could be both a safe and feasible option for HLW disposal, and called for more research into the solidification of HLW which mostly took a liquid form at the time.^17^ On top of this, in the case of GD, the HLW was to “disposed of without concern for its recovery” (p. 86). As such, confidence that HLW could be safely contained and disposed of in the near future was established by the Committee’s research.

### 1957-Present: The Path to Lock-in

This promise of GD as a passively safe future solution for HLW disposal provided the nascent nuclear energy industry with the reasonable assumption of manageable long-term safety, which was important given the risks involved. On top of this, the dominant assumption from the late 1950s until the mid-1970s was that SNF from the civil nuclear energy program would be reprocessed to extract fissionable uranium and plutonium, which would be reused for further energy production^18^ (Walker 2009). As such, the future development of both a civil reprocessing industry as well as GD facilities was considered a sufficient and realistic HLW management strategy. Nuclear authorities stood by the idea that the problem of radioactive waste was technically soluble (U.S. Atomic Energy Commission 1962 p. 55). Also, based on a series of hearings by the Joint Committee on Atomic Energy in 1959, the authorities were convinced that the radioactive waste problem should not slow down the development of the nuclear energy industry and that it would be possible to protect the public during this development (Metlay 1985 p. 236). This confirmed the AEC’s confidence in the possibility of safe radioactive waste management and its prioritisation of industrial promotion over HLW management. This attitude endured for over a decade despite a number of incidents at early above-ground HLW storage sites between 1959 and the mid-1970s (Metlay 1985), which nonetheless spurred the adoption of additional safety features in the 1960 and 1970s such as multi-layered storage casks for HLW and the solidification of HLW where it had been liquid before^19^ (Metlay 1985). As such, additional steps were taken towards the greater capacity to *contain* HLW and its risks.

While the 1960s saw an exponential increase in orders for nuclear power plants, serious practical research into GD was also being undertaken by the AEC, and in 1966 a follow-up committee reaffirmed the conclusions of the 1957 report that GD was the most promising solution for the disposal of HLW (National Research Council 1966). Moreover, the civil reprocessing industry saw its humble beginnings (heavily promoted by the AEC) with the start-up of the reprocessing facility at West Valley, New York in 1966. With a second plant at Morris, Illinois and a third at Barnwell, South Carolina receiving construction permits in 1967 and 1970 respectively, the development of the civil reprocessing industry had apparently been kick-started (Metlay 1985).

In 1970, Lyons, Kansas was proposed as the site for the very first full-scale GD demonstration project.^20^ This decision was supported by further research by the National Research Council that confirmed Lyons’ adequacy as a pilot facility site and again stressed GD’s appropriateness for HLW disposal (National Research Council 1970). However, not everyone shared the AEC’s optimism about the safety and appropriateness of the site [there were numerous boreholes present due to earlier explorations for oil and gas and some water migration could not be properly accounted for (Metlay 1985)], and the proposal was dropped 2 years later for technical and political reasons. Despite this setback, the AEC still pushed for an expansion of the geological disposal program, extending the search for other possible sites for GD. Nonetheless, in the wake of the difficulties with the Lyons site, and as public opposition to nuclear energy was picking up in the early 1970s, other possibilities for HLW management were considered (Vandenbosch and Vandenbosch, 2007). Firstly, an attempt was made by the AEC to implement Retrievable Surface Storage Facilities as a possible medium-term solution for HLW. This proposal was rejected by opponents, including the public, politicians and the Environmental Protection Agency (EPA, set up in 1970), partly out of fear that these facilities would become low-budget permanent solutions (U.S. Congress Office of Technology Assessment 1985). It was subsequently dropped in 1975. Secondly, several options for the final disposal of HLW were further investigated and compared, like extra-terrestrial disposal, disposing of waste in the seabed, in or under ice sheets in the Arctic, transmutation of certain waste types and indeed, GD (e.g., U.S. Atomic Energy Commission 1974).^21^ On top of these difficulties for the GD program, the reprocessing industry was not at all thriving in the way the AEC had hoped. The West Valley plant stopped operation in 1972, when modifications to solve operational and environmental regulatory issues were deemed uneconomical. The Morris plant, finished in 1974, never came into full operation due to technical problems and equipment failures and was abandoned in the same year. Finally, the Barnwell facility was meant to start operation in 1974, but construction delays and licensing issues prevented that deadline from being met (Stewart & Stewart 2011). In short, the AEC’s plans for HLW management were not running smoothly.

Not only HLW management was in some trouble around this time: the nuclear energy industry had to learn the hard way that the optimism about atomic energy “too cheap to meter”^22^ was sorely misplaced, especially as the AEC was obliged to enforce stricter regulations on the industry under growing pressure from environmental groups and the EPA. As such, orders for power plants dropped significantly. This same pressure laid bare the conflict of interest the AEC operated upon (promoting as well as regulating the nuclear energy industry), which led to the AEC being disbanded by the Energy Reorganization Act in 1974, its responsibilities split between the Energy Research and Development Administration (ERDA; promotion) and the Nuclear Regulatory Commission (NRC; regulation, licensing, materials management and setting of safety standards)(Stewart and Stewart 2011). This led to even stricter regulation, which increased costs and made it even more difficult to get licenses for nuclear power plants (Clarfield and Wiecek 1984). As the expansion of nuclear energy production capacity was slowly grinding to a halt in the latter half of the 1970s, the societal pressure that previously led to the disbanding of the AEC rekindled critical attention as well as urgency for HLW management. So despite other options at least being investigated, ERDA continued the AEC’s quest for the expansion of GD with the National Waste Terminal Storage (NWTS) Program in the latter half of the 1970s, wanting to build six repositories by 2000. In light of these developments, however, that period also saw increased critical input from geologists and physicists on GD’s feasibility. The optimism that generally governed the AEC’s attitude towards GD now met with more critical inquiry, which was reflected in the Interagency Review Group on Nuclear Waste Management’s (1978) report to the US president. The report acknowledged that knowledge, experience and predictive capability on repository operation was lacking. And while it still strongly recommended proceeding with GD, it also advised using a “technically conservative” approach (e.g., p. 46), which includes *reversibility* of waste emplacement decisions (p. 18) and temporary *retrievability* of emplaced high-level waste during an initial period of repository operation (e.g., p. 46).^23^ Other developments helped increase the USA’s dependence on GD, as the closed fuel cycle that the AEC had pushed for two decades was plagued with even more difficulties. While the newly founded NRC was investigating the proliferation concerns connected to plutonium recycling and the safeguards necessary to make it work in 1975–76 (which worried a nuclear energy industry that still favoured reprocessing), reprocessing received increased public attention (Walker 2009). This escalated when reprocessing became a prominent theme in the presidential race between President Ford and Jimmy Carter, in which both eventually expressed reservations with regards to the appropriateness of reprocessing SNF. After Carter became president, he issued a statement (Carter 1977) that the USA would “defer indefinitely the commercial reprocessing and recycling of the plutonium produced in the U.S. nuclear power programs”, and that “a viable and economic nuclear power program can be sustained without such reprocessing and recycling”. Official policy turned against reprocessing and the Barnwell reprocessing facility was mothballed and never came online, which effectively meant the end of the civil reprocessing industry.^24^ So not only was GD the only technology of the AEC’s old program that had any promise of becoming a reality, but without reprocessing of SNF the U.S. nuclear fuel cycle would generate larger quantities of high-level waste that remain radioactive for significantly longer than in a fuel cycle with such reprocessing (Taebi and Kloosterman 2008), since it would have to dispose of unprocessed SNF. As such, it became even more critical to look for high-level waste management technologies that were focused on maximal long-term safety, something GD was already known for. From this point on, there was little question as to which technology would be best for the management of high-level waste [as it was in the mid-1970s (e.g., U.S. Atomic Energy Commission 1974)], despite the fact that not reprocessing SNF put more severe demands on repository design and siting.

Implementation of GD still proved difficult though, as the search for possible sites in light of the NWTS met with many negative reactions from state executives and lacked permissions for exploration. Combined with federal budget cuts, this forced the geological disposal program to forego the desired expansion. Nevertheless, efforts to operationalize GD continued. Shortly after the publication of the abovementioned IRG report, the DOE (formerly ERDA) published its Generic Environmental Impact Statement on Commercial Radioactive Waste Management in 1980, which was intended to support a programmatic decision to focus efforts on mined GD (Metlay 1985). Around the same time, the NRC was working on its proposal for the technical criteria that should govern repository licensing,^25^ also focussing on GD as the standard solution (Walker 2009). This coalescence of institutional efforts towards the implementation of GD was subsequently expressed in the 1982 Nuclear Waste Policy Act (NWPA), which followed the DOE’s and the NRC’s commitment to mined geological disposal. Moreover, the act added even more urgency into the equation by aiming for repositories to be operational by 1998 (and capable of taking unprocessed SNF), and shifting some focus away from Monitored Retrievable Storage^26^ (MRS, similar to the AEC’s Retrievable Surface Storage Facilities), saying it was not a complete alternative to GD (Vandenbosch and Vandenbosch 2007). On top of all this, the government would provide only limited support for temporary storage as it could be perceived as a reason to delay final disposal efforts. Following the establishment of the 1982 NWPA, nine sites were selected as possible candidates for repository construction. In the following years, a complex process of negotiations narrowed this list down to three: Hanford, Washington; Deaf Smith County, Texas; and Yucca Mountain, Nevada. However, partly driven by political and cost considerations, the search was even further narrowed down in the 1987 Nuclear Waste Policy Amendments Act, limiting site characterization efforts to Yucca Mountain, Nevada only.

Yucca Mountain’s history is interesting in its own right,^27^ as it has been central to decades of struggle to construct a working GD facility. However, I think it unnecessary to elaborate on it here, for two reasons. Firstly, the analysis as presented above contains the necessary elements for explaining GD’s rise to dominance and why it could be difficult to do otherwise (see “Is Geological Disposal Locked-in?”). Further describing the case of Yucca Mountain and the policy-making around it would not take the analysis in a significantly different direction. Secondly, the case of Yucca Mountain and the adherence to GD even after Yucca’s failure arguably serves better as evidence for GD’s tenacity rather than as an explanation for it (aside from increased commitment and added urgency factors which were certainly not absent before). Indeed, due to significant technical as well as social and political hurdles, Yucca Mountain never became the USA’s first non-military GD site. In 2011, the Obama administration even gave up further efforts to make it into a working disposal site for SNF, as such eliminating hope of having an operational repository in the near future. However, in spite of a history riddled with difficulties (of which three decades revolved around Yucca Mountain), GD remains the go-to option for high-level waste management in the USA (e.g., Blue Ribbon Commission on America’s Nuclear Future 2012).

### Is Geological Disposal Locked-in?

After all this, is GD locked-in in the USA? Let me start by discussing two objections to the idea that this is even possible, or that we can know that it is so.

First, one could question how it could be possible for GD to be locked-in if it seems incapable of actual implementation, even after decades of effort. However, as argued in “Path Dependence and Lock-in”, if symbolic and institutional micro-irreversibilities are sufficient to drive actors to continuously commit to a specific technology, this could be all that is necessary for that technology to be locked-in. At least, it could be enough to make the process of technology development and implementation inflexible in terms of the practical possibility of changing its course, i.e., path-dependent. In other words, having many material manifestations does not make a technology irreversible; having the relevant actors repeatedly orienting their actions towards making that technology (even more of) a reality does.^28^ The way this worked out in the case of GD is summarized below.

Second, can we know if GD is locked-in if no ‘realistic’ alternatives are currently available to which one could switch? After all, in many famous (albeit not uncontroversial) cases of path dependence and lock-in, equally good or even better alternatives were available but not being selected, for example with VHS tapes (Arthur 1990), the QWERTY keyboard (David 1985), or PWR reactors (Cowan 1990). Would most actors still commit to GD if a better solution was available? Unfortunately, this is a counterfactual that is impossible to prove. As such, it would seem at first glance that any claim that GD is irreversible can only be trivially true, i.e., it is impossible to switch to an alternative as long as there are none. This, however, neglects three factors. One, what counts as an equally good or better alternative is not set in stone. That safety and containment have long been leading in the judgment that GD is the only realistic path to follow is to some extent historically and politically contingent. Two, it is possible to add plausibility to the claim that GD is locked-in by showing that its history exhibits characteristics of a path-dependent process, i.e., micro-irreversibilities driving increasing returns in favour of GD, leading up to a point at which it is difficult to do something other than GD. Three, the fact that no realistic alternatives are available at this point in time partially follows from the very historical developments that lead to GD’s dominance. All three factors are discussed in this section.

Already gaining salience during GD’s genesis before 1957 and inspired by the post-WW2 period, the themes of safety and containment have since guided the management of HLW and SNF. As such, these themes have been increasingly embodied materially (e.g., solidification of liquid HLW, multi-layered storage containers, and of course, the technology that is GD) and institutionally (e.g., the separation of the military and civil nuclear energy program, Carter’s decision to refrain from reprocessing to contain the atom’s proliferations risks, the urgency in the NWTS and NWPA for curtailing above-ground SNF build-up and continuous institutional commitment to GD as a way of doing so). In turn, these embodiments have helped reinforce and operationalize safety and containment as leading values. As such, the adoption and continuous reaffirmation of these values functioned as symbolic micro-irreversibilities that supported the path of GD as an appropriate solution for HLW and later, SNF.^29^

As GD’s story unfolded after its contingent starting period (1944-1957), an accumulation of micro-irreversibilities occurred favouring GD. These, combined with broader societal developments, have repeatedly helped drive actors to adhere to GD as the final solution for HLW and SNF. Indeed, after the themes of safety and containment gained prominence and the 1957 Committee on Waste Disposal report proposed GD as the most promising method for making them a technological reality, GD received the institutional commitment of both the AEC and the industry (albeit in combination with reprocessing of SNF). GD was now embedded as an essential part of policy for future HLW management. During the 1960s, serious research into GD (including small-scale test sites) acknowledged its feasibility as well as increased its lead compared to alternatives, which were not systematically looked into since optimism concerning GD’s appropriateness and feasibility was wide-spread. However, as GD came closer to real implementation it ran into difficulties (exemplified by the failure at Lyons, Kansas), as did the organisation responsible for it: the AEC. The AEC was disbanded out of worry about the conflict of interest it operated upon, and alternatives for GD were more systematically investigated. However, several factors kept GD on its dominant course. Firstly, while actors were more critical of GD during this time, the value system behind its selection was not under similar scrutiny. Secondly, the pressure on the nuclear energy program to urgently provide solutions was significantly increased by a number of factors: the end of reprocessing and the fact that now SNF needed to be disposed of, Carter’s strong political stance on the dangers of proliferation combined with increasing SNF build-up, increased societal displeasure with the nuclear energy industry, and the failure to implement a temporary arrangement in the form of the Retrievable Surface Storage Facility. It is unsurprising, then, that the response to critical inquiry into GD in the late 1970s actually was greater commitment to GD under an increased sense of urgency. Like when PWRs were selected for power generation (Cowan 1990), urgency can be an important driver for conservatism in technology selection. What was needed was a technology with which there was considerable experience, even if there may have been alternative technologies for the job eligible for (further) development. Thirdly, ERDA continued the AEC’s quest for expansion of the GD program, assuring continuity of institutional commitment. As a result of all this, GD survived its minor 1970s crisis. After this point, GD’s practicability (with increased knowledge, experience, and increasingly structured institutional frameworks) and political legitimacy (with the explicit commitment to GD in the 1982 NWPA) further increased, as such making it even more into the ‘realistic’ solution it is still taken to be.

In addition to these mechanisms supporting GD, there were also reasons why alternative paths were specifically not selected. For example, in a situation of limited resources available for organizing high-level waste management (especially at a time when the focus was on developing the energy industry rather than on ways to manage its wastes properly), it is clear that commitment to GD would mean even more limited resources available for development of possible alternatives, especially when it is assumed that there is little reason to do so. Indeed, until the mid-1970s, the AEC and the industry saw little need to systematically look into and develop alternatives to reprocessing and GD. Some possible alternatives, like disposal in the seabed or under Arctic ice sheets, would have also been unpopular both with an increasingly environmentally aware public in the 1970s as well as other countries across the world. Also, further development of more advanced fuel cycles that would reduce waste lifetime (and as such, lessen demands on disposal technologies) were incompatible with the ban on reprocessing in 1977 as they were judged to give rise to unacceptable proliferation concerns.

After all this, the case of Yucca Mountain, its failure, and the subsequent retention of GD as the most favourable solution for high-level waste disposal attests to the fact that a point has been reached at which switching to an alternative solution for high-level waste management has become difficult (not least because possible alternatives, other than temporary storage, are underdeveloped). Still, this is quite peculiar given the lack of working civil GD sites in the USA.^30^ Apparently, it can become extremely difficult to change course on the choice for a specific technological solution despite extremely few actual working instances of the technology itself.

Finally, allow me to briefly elaborate on the evolution of reversibility considerations in GD over the course of its history. It is interesting that while the National Research Council’s 1957 report contends that HLW should be emplaced in geological repositories without concern for its retrieval, the 1979 IRG report features provisions for limited retrievability on the basis of epistemic and prudential considerations. This was both politically salient as well as in line with the critical appraisal of GD in the late 1970s. And while the NEA’s *reasons* for retrievability presented in “Reversibility as an issue in radioactive waste management” have significantly expanded in scope to considerations of justice when compared to the IRG’s, the *practical* side of reversibility and retrievability does not seem to have followed suit. Indeed, while the reasons for reversibility considerations have significantly evolved, our choice and design of the technology meant to fulfil these has not sufficiently done so, as evidenced by the discrepancies also presented above. On the one hand, if GD is locked-in, this could possibly help to explain why these discrepancies exist between the NEA’s reasons for reversibility and retrievability and the extent to which GD seems to be an appropriate means of achieving them, since it would be extremely difficult to change to a solution more in line with new reasons for wanting reversibility. On the other hand, the inclusion of reversibility and retrievability considerations in GD does not seem to have lessened its dominance. *Au contraire*, making GD compatible with increased demands on high-level waste management [be it epistemic demands (IRG) and/or demands for justice (NEA)] would make it less pressing to work towards alternatives. So the inclusion of reversibility considerations, while lowering the probability of problems with GD arising, has not alleviated GD’s lock-in.

The history of GD sketched above contains ample micro-irreversibilities that would lead GD to become locked-in by making it more likely that agents favour GD. By the same token, and partly due to the same developments that led to GD’s dominance, alternatives have not been extensively pursued. So, in addition to GD being locked-in in a trivial sense (no ‘realistic’ alternatives are currently available), these factors provide plausibility to the idea that GD is locked-in due to being unable to shake free from its own history. As such, considering the first question put forward in “Reversibility as an issue in radioactive waste management”:

- Would an authorized body be reasonably able to switch from GD to an alternative solution if problems with GD were to arise?,

it seems that, at least for the USA, one would have to conclude that it would be at least difficult and at worst impossible for an authorized agency to step down from GD as the dominant high-level waste management technology, at least within a reasonable timeframe. Given that GD is the preferred solution to the high-level waste problem in most nuclear energy-producing countries,^31^ and that other countries do not have access to more alternatives to GD than the USA does, I think it not unreasonable to expect that in some of these countries, GD might be similarly locked-in.^32^

If all the above holds true, GD at least partly fails to meet one of the conditions and can thus not be considered a truly reversible technology (in those specific cases). However, one could ask whether GD’s lock-in is really problematic, given that (a) scientific confidence in the capacity of engineered barriers and geology to contain high-level waste is significant, and (b) that no technology is readily available on a satisfactory scale to turn high-level waste into benign substances? That is, is it not a good strategy for ‘undoing’ the morally undesirable consequences of nuclear energy technologies? This question relates directly to the second question put forward in “Reversibility as an issue in radioactive waste management”:

- Does GD exhaust the possibilities of undoing the consequences connected to high-level waste, and the hazards that could come about due to the use of GD for managing this waste?

In the following section, I contend that there are different general strategies for undoing such consequences that one can follow in developing a technology, and that some are preferable over others, at least *qua* reversibility. GD is principally focused on one of these strategies, albeit not the most preferable one.

## On Geological Disposal’s Capacity for Undoing Consequences

What does it mean to ‘undo the consequences connected to high-level waste’? What would constitute an ‘ideal’ undoing of consequences is, practically speaking, impossible: one simply cannot go back in time and start over. Nevertheless, what sorts of action could one still undertake towards the undoing of consequences, limited as they may be? In what follows, I present four practical strategies for ‘undoing consequences’ in order of decreasing similarity to ‘ideal’ undoing:

1. *Remediation* bringing (parts of) the system under consideration back to a previous state by eliminating the problem source and using (part of) the system’s internal dynamics to undo the unwanted effects of the technology’s development and implementation. This seems to require the least invasive effort, and leaves a solid basis for other developments.
2. (*Re*)*construction* bringing (parts of) the system under consideration back to the state by eliminating the problem source and actively reconfiguring system parts to reconstruct the previous state so as to undo the unwanted effects of the technology’s development and implementation.

Note that the previous two imply elimination of the problem source. In the case of RWM in general and of GD in particular, high-level waste would have to be considered the most important ‘problem source’, and this is what the rest of this section will focus on. Other possible problem sources might be specific institutional arrangements or possibly outdated value systems (i.e. institutional or symbolic elements mentioned as micro-irreversibilities above). Given this possibility, undoing certain consequences may be as ‘simple’ as reverting to a state in which multiple possible paths were open, i.e. getting rid of lock-in. However, there are two more strategies for undoing consequences, ones in which the problem source is not eliminated:

3. *Containment* Containment of the problem source without eliminating it, shielding potential victims from its harmful effects.
4. *Compensation* Compensate victims for the undesirable consequences of the technology development project when even containment not possible.

One important point to make about these strategies is that if one wants to reasonably ensure that these options are available when the need arises, the technology in question needs to be *designed* according to these strategies. Another point is that these strategies are not mutually exclusive, and will most likely have to be used in conjunction. In doing so, there is a preferable order to these approaches: what cannot be solved by remediation should be tackled by reconstruction, etc. In this way, the potential for undoing unwanted consequences is exhausted to the greatest possible extent. These insights do have their implications though, the most important of which is probably the following: already during the development of a technology, one should aim for remediable and reconstructible solutions rather than ones dependent on containment or compensation. From the point of view of reversibility, the latter are little more than ‘end-of-pipe’ solutions necessitated by our incapability to construct more reversible technologies by eliminating problem sources. The question is: which of these strategies does GD exemplify?

One could argue that GD is a technology based on remediation. After all, the internal dynamics of the system (radioactive decay) will eventually undo the unwanted effects connected to high-level waste. When, after thousands of years, the waste reaches the radiation level of natural uranium ore, would the situation not be remediated? Well, at least not in the way that remediation is meant here as a strategy for undoing consequences: remediation would have to include the elimination of the problem source, no active steps towards which are actually undertaken in GD. Charitably to GD, however, one could argue that our actions implementing GD now do eliminate high-level wastes eventually. However, can we then really say that our actions eliminate these wastes? High-level waste and the risks connected to it (while diminished through multiple engineered and natural barriers) exist as possibly problematic for an extended amount of time, one that far surpasses any example of institutionalized practice or organized action. As such, even on this charitable reading GD fails to eliminate the problem source within a timeframe that is relevant for a *practical* conception of remediation as a strategy for undoing consequences. As such, we cannot claim that GD is a remediation-based technology.

Despite appearances (it requires very specialized and scientifically advanced construction after all) GD is also not reconstruction-based for the same reason mentioned above: the high-level waste is just not eliminated quickly (or actively) enough. At most, it could be said that retrievability considerations in GD’s design do allow for some reconstructive action in case unwanted effects do occur, whether these effects are connected to the dangers of radiotoxicity or intergenerational injustice. However, the limited timespan for which retrievability is envisioned, combined with its diminishing nature and the fact that while retrieval would remove the problem source from its location but not entirely eliminate it, leaves GD’s potential for reconstruction rather limited.

In the end, GD corresponds largely to the *containment* strategy: despite limited retrievability provisions, containment is indeed the design goal of GD. Rather than actively eliminating the problem source, it is contained behind multiple barriers, e.g., a vitrification matrix, multi-layer canisters, the repository with its multiple engineered barriers and even stable geological layers. But this is not all. The ‘containment’ strategy is so pervasive in GD that even its institutional and symbolic elements were oriented towards containment, at least for a large part of GD’s history. For example, technocratic elites have generally left little room for public participation in how high-level waste was to be handled,^33^ especially during the early decades of nuclear energy development. Additionally, by viewing this waste in terms of difficult-to-control and largely irreversible risks, legitimation of technical and passively safe solutions was assured (especially when combined with a general distrust of social solutions). In short: GD works according to containment all the way down, from the top echelons of nuclear policy making to hundreds of meters below the earth’s surface.

In GD’s defence, however, one might rightly bring up the point that eliminating high-level waste is currently practically impossible. No technology is actually available to turn such waste into benign substances. Given this fact, is GD not the best technology available for taking our responsibility towards future generations? Two points need to be made in response to this. First, while it is true that no technology is currently able to ‘eliminate’ high-level waste, this does not mean such technologies are not at least realistic. For example, a process called partitioning and transmutation (P&T) is being developed which could theoretically decrease total high-level waste volume as well as limit its lifetime to as little as 500–1000 years (Condé et al. 2004). This would constitute at least a partial elimination of the problem source and as such, could be part of a reconstruction strategy to undo the unwanted effects of high-level waste. While a fuel cycle including P&T would be more expensive than using a more traditional fuel cycle, and comes with its own security and safety concerns, it would at least be a step up in terms of undoing unwanted consequences in the form of long-term risks of high-level waste radiotoxicity (Taebi and Kadak 2010). The second point to be made concerns the manner in which containment of high-level waste is achieved in GD: it prohibits or at least makes it incredibly arduous to switch to a more reversible strategy in the future due to a lack of retrievability. For example, by the time P&T would actually be available on a large enough scale to make a significant difference, repositories could be largely or completely closed. And even if retrievability was fully implemented and maintained, the possibility of reprocessing/recycling/destroying some high-level waste would prove difficult, e.g., due to being stabilized in glass or concrete. Indeed, it would seem that the epitome of containment entails closure, not only of repositories and institutional orders, but also closure of different options for switching strategies for undoing (the effects) of high-level waste.

Let me make two qualificatory notes. First, a reconstruction-based technology like P&T is not likely to become a complete replacement of GD, or the strategy it represents. With HLW that remains radioactive for ‘only’ a couple of thousands of years, decent containment would still be necessary. As such, the containment strategy still has a place in the management of high-level waste, but only insofar as reconstruction’s potential has been exhausted first. Secondly, these new circumstances might open up options for the form a containment strategy/technology may take, possibly loosening the lock-in of GD as the dominant final solution for high-level waste management. However, P&T’s infrastructural and institutional demands may institute their own path-dependent processes and possibly locked-in technologies, and have their own negative consequences other than long-term risks of radiotoxicity. In other words, optimizing one of the conditions for reversible technologies might entail losing out on the other. Moreover, since, the operationalization of the two conditions do not allow for a comparison on one similar measuring scale, balancing the two conditions would likely need a careful exercise in practical and/or political reason.

There is currently some attention for the role of *compensation* in RWM (e.g., Kojo et al. 2013). While not usually linked to the reversibility debate outside of a public demand for retrievability provisions, I hope that the four strategies presented provide a clue as to how compensation features in reversible GD. It is a possible strategy for achieving more capacity of undoing unwanted consequences, but only to be applied when the other three are sufficiently exhausted. Although there may be other principal and practical reasons like justice or social acceptance to resort to compensation outside of reversibility considerations, any claim to increased reversibility directly because of compensation should be treated with caution.

To conclude, it seems that the second reason (next to being significantly locked-in) that GD, even with reversibility provisions, apparently fails to meet the NEA’s own justification of reversibility is that its strategy for undoing unwanted consequences is less-than-ideal for two reasons. Firstly, a remediation- or reconstruction-based technology would at least in principle be more able to live up to the NEA’s justification of reversibility. Secondly, the way GD embodies containment is so severe that it disallows remediation or reconstruction of geologically disposed high-level waste at a point in the future at which these options would become viable.

## Conclusion

At the start of this paper, the way reversibility features in GD was explored, and a number of critical discrepancies were noted between the reasons given for the inclusion of reversibility provisions in GD and GD’s ability to live up to these reasons. This prompted the question whether GD could be considered a reversible technology, since such a reversible technology would arguably be able to fulfil the reasons given in “Reversibility as an issue in radioactive waste management”. It was then put forward that for GD to be considered reversible, two questions need to be answered affirmatively:

- Would an authorized body be reasonably able to switch from GD to an alternative solution if problems with GD were to arise?
- Does GD exhaust the possibilities of undoing the consequences connected to high-level waste, and the hazards that could come about due to the use of GD for managing this waste?

Considering the second question, it was found that GD’s strategy for undoing high-level waste-related consequences was less-than-ideal, since it relies mainly on a strategy of containment rather than on eliminating the waste through reconstruction or remediation. And while it is true that no technology exists that embodies these more ideal strategies, the way GD’s containment works makes switching to these strategies for existing wastes rather difficult. Still, if and when such a technology eventually becomes available, could we not simply switch to it? The answer to the first question gives us reason to worry about this possibility. It would appear that GD is currently locked-in in the USA and quite possibly in other countries that espouse GD as well. The historical process of GD’s development and operationalization has brought us to a point where these societies’ symbolic, institutional and material investment in GD makes it difficult and thus unlikely that GD will be replaced with an alternative any time soon. With *at least* one of the questions answered negatively the conclusion follows that despite laudable efforts to the contrary in the past decades, GD is currently a practically irreversible technology for RWM. Taking measures both to avoid lock-in situations as well as exhausting the (future) possibilities for reconstruction and remediation for undoing high-level waste-related consequences could be fruitful strategies for increasing RWM’s reversibility. However, optimizing both of these conditions of technological reversibility might prove difficult with some technologies, since scenarios are imaginable in which solutions in favour of one of the conditions decrease the potential of the other. For example, the future introduction of P&T could improve the ability to undo high-level waste-related consequences (since it is a reconstruction-based technology) and even lessen the severity of GD’s lock-in (if applicable). On the other hand, P&T relies on extensive and complex infrastructures which could themselves become locked-in and have their own undesirable consequences. This creates an additional difficulty for finding truly reversible technologies or for balancing the two aspects in a way that is satisfactory, since an affirmative answer to both questions is necessary to truly speak of technological reversibility.

Of course, it must be remembered that this paper is focussed specifically on reversibility. However, when deciding on the specific form an RWM technology is supposed to take, or even which one(s) to select, more values are bound to be eligible for serious consideration. Indeed, issues of safety, justice, feasibility, efficiency, etc. also need to be considered, and might turn out to be partly incommensurable with a technology’s reversibility. As such, this paper is not meant as a plea for the sole consideration of reversibility in the GD debate. Rather, it provides a clarification of what technological reversibility entails and how it is to be achieved, which is essential if reversibility is to be considered next to other important values.

What does all this mean for the hypothetical experimenter the paper opened with? After all, if she is to be prepared for learning that the experiment is to be stopped, the technology she is experimenting with should in principle be reversible. If the analysis presented in this paper is correct, reversibility can only be ensured by its proactive consideration, both in designing the nuclear energy technology in question (according to strategies for undoing undesirable consequences) as well as keeping alternative solutions viable and avoiding disproportionate institutional and symbolic commitment (avoiding lock-in). This would mean that GD’s lock-in as well as its less-than-ideal prioritization of the containment strategy require careful revision. As it stands, however, the inclusion of reversibility in GD by the NEA is hardly adequate to the reasons provided for it, let alone to the standards of properly reversible experiments with RWM technology.

Lastly, to what extent do the results of this case-specific analysis carry over to the general framework of social experimentation with new technologies? While the recommendations for avoiding lock-in could work for other technologies due to their generality, the strategies for undoing consequences might need reconsideration. That is, some technologies may have different options for undoing consequences which invite different strategies for doing so, although this would have to be determined on a case-by-case basis. The original phrase by van de Poel “containment of hazards as far as reasonably possible” (2011 p. 289) turns out to be overly specific in this regard, since containment is just one possible strategy for undoing undesirable consequences. It must be noted, however, that responsible social experimentation demands more of a responsible experiment than it simply being reversible (van de Poel 2011). So, while (some) reversibility might be a necessary condition of responsible experimentation, it is by no means sufficient.

## Footnotes

1 The development of Probabilistic Risk Assessment (PRA) is especially noteworthy here, pioneered in the famous WASH-1400 or Rasmussen Report (U.S. Nuclear Regulatory Commission 1975).

2 In 1953, Obninsk, Russia was home to the very first reactor providing power to the national grid. As this was mainly a research reactor, however, its power output was rather limited. By 1956, Calder Hall, United Kingdom housed the first large-scale nuclear power plant, although it also produced plutonium for the British military program. In 1957, the Shippingport Atomic Power Station in Pennsylvania became the first large-scale nuclear power plant that was fully devoted to civil nuclear energy production.

3 These are the two conditions that are most relevant to considerations of reversibility out of a longer list by van de Poel (p. 289).

4 That is, the websites of the U.S. Nuclear Regulatory Commission (NRC), the International Atomic Energy Agency (IAEA), the OECD Nuclear Energy Agency (NEA), the French National Radioactive Waste Management Agency (ANDRA), the Swedish National Council for Nuclear Waste (KASAM), and the International Commission on Radiological Protection (ICRP) were searched for the terms ‘reversibility’, ‘reversible’, ‘irreversibility’, and ‘irreversible’.

5 This portrayal of the distinction does not include exempt waste, very short-lived waste, and very low-level waste, since they require relatively little or short duration shielding or regulatory control (IAEA 2009).

6 In a fuel cycle without reprocessing of SNF (the ‘open’ fuel cycle, e.g., in Canada, Sweden, and the USA), SNF is considered high-level waste when it is accepted for disposal. In a fuel cycle with reprocessing of SNF to extract uranium and plutonium for recycling (the ‘closed’ fuel cycle, e.g., in France, India, and Japan), high-level waste from nuclear energy production consists mainly of the fission products left over from this reprocessing (IAEA 2006), which are normally solidified before disposal. This distinction between HLW from reprocessing and SNF without reprocessing is highly significant: while unprocessed SNF has a waste lifetime of about 200,000 years, reprocessing can reduce high-level waste lifetime to about 5000 years (Taebi and Kloosterman 2008).

7 Per definition, in the case of *storage*, retrieval of the waste is envisioned for some point in the future, whereas *disposal* implies emplacement of waste without the intent of eventual retrieval.

8 In line with the distinction given above, I use the formulation ‘high-level waste’ to mean the category as defined by the IAEA (2009), which in the case of nuclear energy production includes both SNF and HLW as presented in footnote 6.

9 Note that this does not mean that actual confinement of radionuclides is guaranteed indefinitely (which is technically impossible), just that the timescales involved prescribe extremely long-term emplacement.

10 For a brief discussion of the evolution of reversibility provisions in GD in the USA, please see Sect. 3.4.

11 Not only could this happen due to technical difficulties, but also through learning about what we should or should not consider ‘apt’.

12 If it is currently impossible to switch to an alternative, then the technology is not currently reversible. Whether or not some future developments might change this situation has little bearing on the technology’s current reversibility status.

13 Initiated in the late 1940s, the nuclear naval propulsion program launched its first PWR-powered submarine—the USS Nautilus—in 1954.

14 This HLW was apparently stored without concrete plans of its further management, an example of the prioritization of the application end of the fuel cycle. A tremendously complex and expensive clean-up operation is currently ongoing at the Hanford site to properly deal with 40 years’ worth of Hanford’s HLW in deteriorating storage facilities (Oregon Department of Energy 2014).

15 For a transcript of the speech, see http://www.iaea.org/About/history_speech.html (Accessed March 29, 2013).

16 What was also contained was the nuclear influence of the USSR. The USA’s nuclear industry development had to be privatized in order to be ideologically in line with the American liberal ideal so different from the USSR’s statist communism.

17 The report mentions that the Commission was “convinced that radioactive waste can be disposed of safely in a variety of ways and at a large number of sites in the United States” (p. 3), adding that the “most promising method of disposal of high level waste […] seems to be in salt deposits” (p. 4). Moreover, it promotes the “stabilization of the waste in a slag or ceramic material” (p. 6) as another promising method, away from the predominantly liquid HLW at the time.

18 There was already significant experience with reprocessing technology in the military program. Moreover, the AEC promoted reprocessing out of concern for uranium supply shortages for the nuclear energy industry. Together with the breeder reactors the AEC was looking into, reprocessing would substantially increase the sustainability of uranium resources (Stewart and Stewart 2011).

19 The 1957 National Research Council report had prompted further research into waste solidification which continued through the 1960s and beyond. In 1970, the AEC proposed new regulations to have liquid HLW solidified 5 years after its generation (Metlay 1985).

20 An abandoned salt mine near Lyons already served as a test site for HLW disposal between 1965 and 1968.

21 Although some of these options had at times been considered, this was the first time they were as officially and systematically compared.

22 This phrase was coined by the chairman of the AEC, Lewis Strauss, in a 1954 speech to the National Association of Science Writers (Strauss 1954). While it has become iconic of the economic optimism at the time concerning nuclear power, it is not to be taken as what was actually considered a realistic cost estimate.

23 Note that the NEA’s R-scale (NEA 2011) provides a specific timeline and a more gradual decline of retrievability than does the IRG, and is more operationalized.

24 Although the Reagan administration withdrew the ban on reprocessing in 1981 (U.S. Congress Office of Technology Assessment 1985), it never became part of official U.S. radioactive waste policy again.

25 These criteria included many concepts still visible in the NEA’s proposal today, like multiple barriers, the validation of models, geological uncertainties, and the problem of human intrusion (Metlay 1985).

26 While industry favoured MRS as a temporary solution, environmental groups again protested it out of fear of MRS facilities becoming *de facto* permanent disposal sites. The NWPA only foresaw inquiry into the need for and feasibility of MRS, but did not order any concrete construction (Vandenbosch and Vandenbosch 2007).

27 For a comprehensive overview of the policy and technical difficulties in SNF management in this period, see Vandenbosch & Vandenbosch (2007) and Macfarlane & Ewing (2006).

28 First, note that having many material manifestations can indirectly increase lock-in, since it allows for learning effects, economies of scale, sunk costs, etc., all of which can push actors to commit to the technology, i.e., constitute increasing returns. Secondly, this means that one should not ask whether it is *either* high-level waste policy practice *or* the technology of GD that is irreversible, since policy maker’s continuous commitment could theoretically be sufficient to make GD locked-in.

29 This is in no way supposed to be a polemic against considerations of safety. However, in light of a demand for reversibility, even these value judgements should be open for reconsideration, since other values might prescribe other solutions. For example, an assessment based on maximizing future generation’s opportunities for making use of SNF (something the NEA mentions as one of its reasons for reversibility in GD), would likely select a different solution than GD.

30 For HLW from the military nuclear program, there is the Waste Isolation Pilot Plant (WIPP) in New Mexico which has been receiving waste since 1999, although its history since 1973 has also not been without both technical and socio-political difficulties.

31 One should not forget the impact of international organisation and cooperation. For example, given that the IAEA was set up in 1957 (pushed by the Eisenhower administration after the 1953 ‘Atoms for Peace’ speech), one can imagine the subsequent international spread of the themes of containment and safety (e.g., IAEA, 1956).

32 However, even if this expectation is reasonable, any claim to a specific country having GD as a locked-in technology would have to be backed up by the necessary socio-historical analysis.

33 Given the difficulties to find public acceptance for waste storage sites or geological repositories, these processes have gradually opened up to some extent (Bergmans 2008; Richardson et al. 2011).
